# Vissage percutané du scaphoïde carpien par vis de Herbert - à propos de 10 cas

**DOI:** 10.11604/pamj.2013.14.112.1921

**Published:** 2013-03-22

**Authors:** Najib Abbassi, Najib Abdeljawad, Moncef Erraji, Rachid Abdelillah, Abdelkrim Daoudi, Hicham Yacoubi

**Affiliations:** 1 Service de chirurgie orthopédique et traumatologique, centre hospitalier d'Oujda, Oujda, Maroc

**Keywords:** Scaphoïde, ostéosynthèse, percutanée, vis de Herbert, scaphoid, osteosynthesis, percutaneous, Herbert screw

## Abstract

Les fractures du scaphoïde carpien sont de diagnostic et de traitement difficiles. Les auteurs rapportent les résultats du traitement de ces fractures par le vissage percutané par la vis de Herbert. Les résultats étaient intéressants vu la rapidité de consolidation et la qualité du résultat fonctionnel.

## Introduction

Le scaphoïde carpien est le plus fréquemment fracturé des os du carpe [1]. Le traitement habituel consiste en une immobilisation plâtrée pendant deux à trois mois imposant au patient un arrêt de travail et de l'activité sportive prolongés. En outre la fixation à ciel ouvert constitue une agression de la vascularisation précaire de cet os ainsi que son environnement ligamentaire [2]. Le vissage percutané du scaphoïde constitue actuellement l'alternative thérapeutique de choix évitant les inconvénients de l'immobilisation prolongée et de la chirurgie à ciel ouvert. Les auteurs exposent une série de 10 cas de fractures de scaphoïde non déplacées traitées par vissage percutané.

## Méthodes

### La série

**Figure 1:**
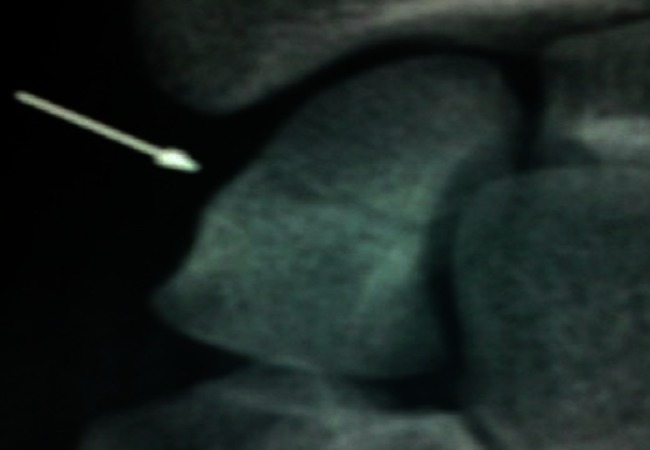
Radiographie du poignet de face centrée sur le scaphoïde objectivant une fracture corporéale non déplacée

Dans une série rétrospective allant de 2009 à 2012, nous avons colligé 10 cas de fractures de scaphoïde non déplacées traitées par vissage percutané. Le sexe ratio est de 9 hommes pour une femme, l'âge moyen est de 28ans (19 à 35). Tous nos patients étaient droitiers et la fracture a intéressé le côté dominant chez 8 patients. Le mécanisme était toujours une chute sur le poignet en extension. Toutes les fractures étaient fraiches avec un délai moyen de consultation de 5 jours (1 à 7). La fracture a été diagnostiquée par un bilan radiologique standard (Figure 1). La classification utilisée est celle de Shernberg, les fractures étaient non déplacées intéressant le corps du scaphoïde: 40% en zone II, 40% en zone III et 20% en zone IV.

### La Technique opératoire

**Figure 2:**
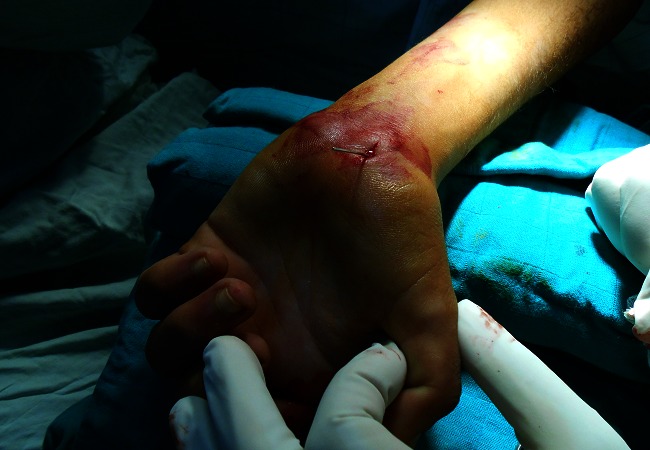
Installation de la main sur scopie poignet en hyperextension

**Figure 3:**
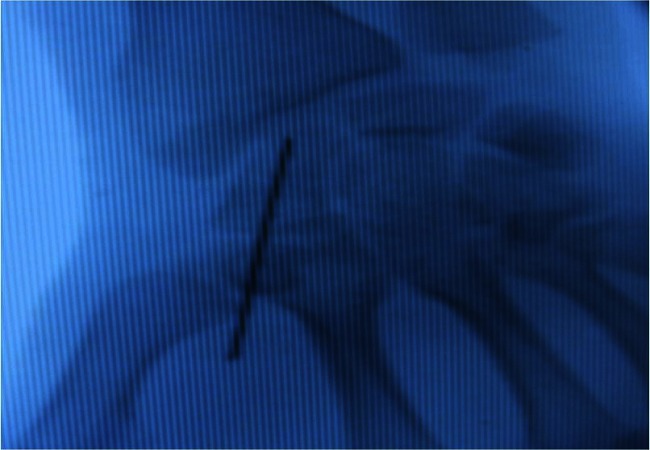
Mise en place de la broche guide qui doit être au centre du scaphoïde et verticale par rapport à la fracture (vue de face)

**Figure 4:**
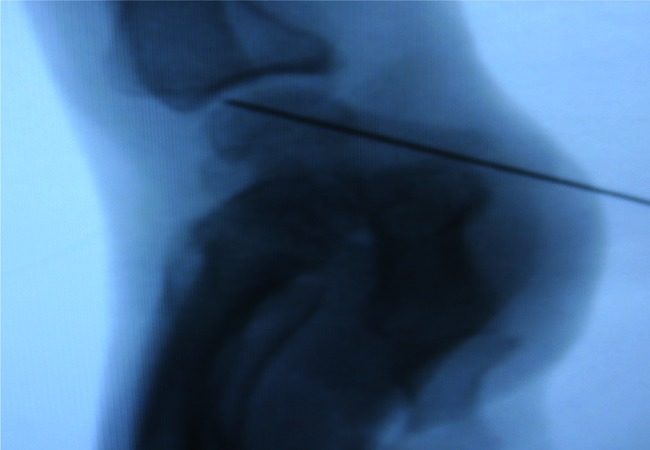
Mise en place de la broche guide qui doit être au centre du scaphoïde et verticale par rapport à la fracture (vue de profil)

Tous nos patients ont été opérés sous anesthésie locorégionale et de manière ambulatoire. La technique opératoire consistait à effectuer, par voie palmaire sous contrôle de l'amplificateur de brillance, un embrochage rétrograde par une broche guide, le poignet étant en extension (Figure 2). La broche était introduite au niveau de la partie externe du tubercule distal du scaphoïde dans l'axe de cet os de face et de profil à la scopie (Figure 3, Figure 4). Cet embrochage est suivi par un méchage puis le vissage. La vis utilisée chez tous nos patients était la vis canulée de Herbert. Les deux pas de la vis ont été de part et d'autre du foyer de fracture et l'extrémité de la vis ne devait pas s'appuyer sur la corticale proximale pour éviter de décoapter le foyer de fracture. Une immobilisation par attelle fut réalisée à but antalgique pendant une semaine pour tous nos patients. Dès l'ablation de l'attelle, nos patients ont été autorisés à reprendre leurs activités habituelles avec restriction des travaux et activités sportives intenses.

## Résultats

**Figure 5:**
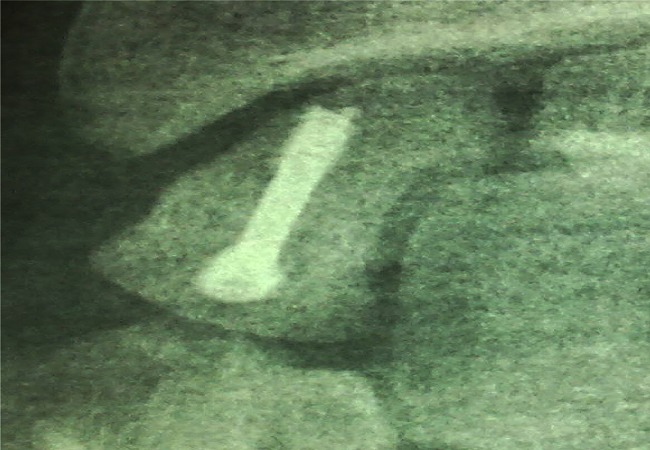
Radiographie de contrôle à la 12ème semaine post-opératoire chez le même patient

**Figure 6:**
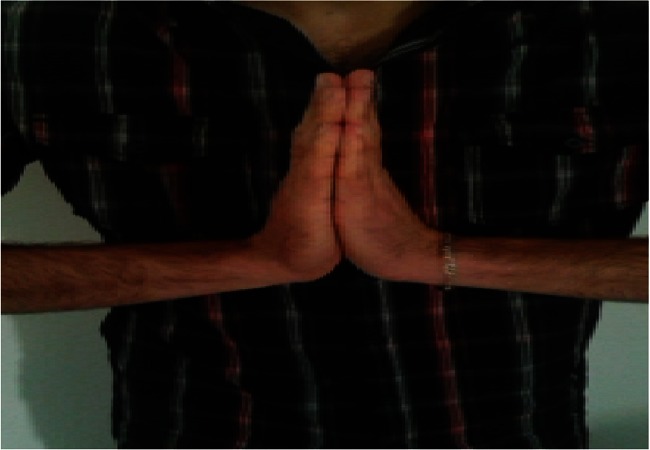
Résultat fonctionnel à 3 mois

**Figure 7:**
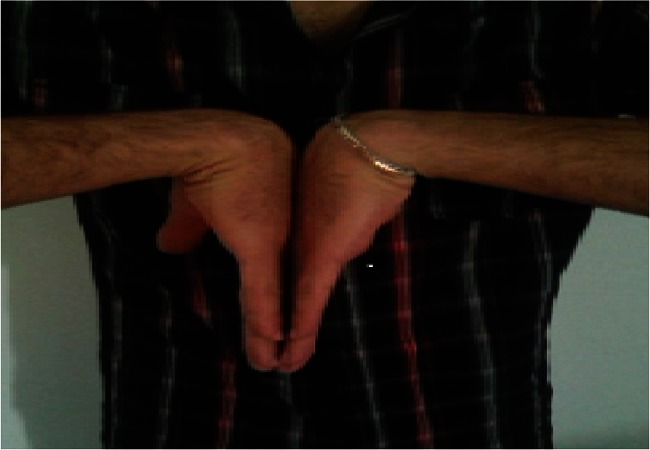
Résultat fonctionnel à 3 mois chez le même patient

Le recul moyen est de 11 mois (5 à 24 mois). Tous nos patients ont été suivis par un bilan radiographique standard effectué chaque mois. La consolidation radiologique a été obtenue chez 100%, entre le deuxième et le troisième mois (Figure 5). Une mobilité sans douleur a été obtenue chez tous nos patients avec en moyenne une flexion du poignet à 71° contre 76° du côté sain, extension à 80° contre 83°, inclinaison radial à 15° contre 17°, inclinaison ulnaire à 29° contre 32° (Figure 6, Figure 7). Tous nos patients ont repris leurs activités professionnelles après 3 mois et ne présentaient pas de douleur à la mobilisation du pouce, ni de perte de force subjective. L'opposition du pouce était au moins de 9 (contact pulpe pouce - pli palmaire inférieur du V) pour tous nos patients sur la cotation de Kapandji.

## Discussion

Le traitement orthopédique des fractures non déplacées du scaphoïde continue à poser encore de nombreuses interrogations: Quelle est la durée minimale d'immobilisation? Faut-il immobiliser le coude? La métacarpo-phalangienne du pouce? Et si oui pour quelle durée? Quelle est la position convenable d'immobilisation du poignet? Quel type de matériau utiliser? Plâtre de Paris, résine, orthèse thermoformée? [3].

Par ailleurs, la chirurgie à ciel ouvert des fractures non déplacées du corps du scaphoïde augmente certes la stabilité, diminue la durée d'immobilisation et donc favorise un retour précoce au travail et au sport sans diminuer le taux de pseudarthrose qui est estimé à 5% [4].

En plus la chirurgie à ciel ouvert expose à certains désavantages: lésions des tissus mous, lésions des ligaments radio-carpiens extrinsèques palmaires ou dorsaux selon la voie d'abord utilisée, dévascularisation du foyer de fracture, raideur du poignet et cicatrice douloureuse [5].

Dans cette optique, le vissage percutané des fractures du scaphoïde offre l'avantage de minimiser les risques opératoires de la chirurgie à ciel ouvert tout en apportant les mêmes bénéfices sur la durée d'immobilisation. La revue de la littérature montre que les différents auteurs partagent cette attitude [6, 7].

La technique est standardisée elle se résume en un abord percutané palmaire du tubercule distal du scaphoïde sous repérage scopique, le poignet étant placé en extension pour ouvrir l'articulation scapho-trapezienne. Ce mini-abord permet de conserver intact l'appareil ligamentaire antérieur du carpe et en particulier le ligament radio-scapho-capital qui joue un rôle essentiel dans le mouvement de bascule du scaphoïde lors des mouvements de flexion et extension et de déviation radiale et ulnaire du poignet [8].

Des études comparatives faites entre l'abord antérieur et postérieur n'ont pas montré une différence significative dans le positionnement de la vis. Par ailleurs le vissage antérograde serait intéressant pour les fractures polaires supérieures [9].

L'étape principale est la mise en place de la broche guide qui doit être au centre du scaphoïde et verticale par rapport à la fracture. Cette broche sert aussi à la mesure de la vis. Certains auteurs recommandent d'utiliser une autre broche dite antirotatoire placée parallèlement à la première, l'intervention se termine par le méchage et le vissage [10].

L'arthroscopie peut être indispensable pour contrôler la réduction et le bon emplacement de la vis mais, elle est aussi utile pour la réduction des fractures déplacées élargissant ainsi les indications du vissage percutané du scaphoïdes aux fractures déplacées [11].

Enfin il faut garder à l'esprit que l'on connait encore mal l'incidence des lésions du ligament scapho-lunaire associées aux fractures non déplacées du scaphoïde et qu'il apparait logique que l'on s'expose à démasquer des instabilités scapho-lunaires à distance d'un vissage du fait de la mobilisation précoce du poignet [10]. Ces mêmes lésions scapho-lunaires auraient sûrement pour la plupart cicatrisé au cours de l'immobilisation plâtrée de plusieurs mois.

L'arthroscopie du poignet per-opératoire prend alors toute son importance en l'absence d'arthroscanner pré-opératoire [8].

Les taux de consolidation sont excellents dans les différentes séries, 100% dans notre série, 90% pour Brutus et al [12], 100% pour Ledoux et al [13], 100% pour Haddad et al [14], 90% pour Resines-Erasun et al [6]. Les complications sont rares il s'agit surtout de torsion ou de bris de la broche guide [11]. Un cas Rupture du tendon du fléchisseur radial du carpe a été rapporté par G. Ducharne et al [15].

Cependant, même en cas de pseudarthrose du scaphoïde, adéquatement vissée en compression, si celle-ci reste complètement asymptomatique et sans lyse radiologique aucune autre intervention ne sera réalisée dans l'immédiat mais un suivi annuel sera nécessaire [8].

## Conclusion

Actuellement le vissage percutané du scaphoïde est l'indication de choix des fractures corporéales non ou peu déplacées. Le taux de consolidation est excellent dans un délai court permettant un retour rapide au travail et à l'activité sportive.
